# A Review and Clinical Understanding of Tenofovir: Tenofovir Disoproxil Fumarate versus Tenofovir Alafenamide

**DOI:** 10.1177/2325958220919231

**Published:** 2020-04-15

**Authors:** Chanie Wassner, Nicole Bradley, Yuman Lee

**Affiliations:** 1 Department of Pharmacy, NYU Langone Hospital, Brooklyn, NY, USA; 2 College of Pharmacy and Health Sciences, St. John’s University, Queens, NY, USA

**Keywords:** HIV, tenofovir, hepatitis B, postexposure prophylaxis, pre-exposure prophylaxis

## Abstract

HIV is a serious chronic medical condition. Significant improvements in antiretroviral therapy have led to a transformation in its management. No curative treatment is available for HIV, and lifelong therapy is required with a combination of agents to control viral replication and prevent complications. Some of the older agents are notorious for many side effects, making patient compliance difficult, which is critical to preventing HIV resistance. Tenofovir is one of the newer, more tolerable, nucleotide reverse transcriptase inhibitors on the market; is a mainstay of many antiretroviral therapy combinations; and is now available in 2 different formulations, tenofovir disoproxil fumarate (TDF) and, the more recent, tenofovir alafenamide (TAF). These 2 formulations have very different pharmacokinetics, which seem to affect their efficacy and safety. This manuscript provides insight into the history of TDF and TAF development, their unique pharmacokinetics and pharmacology, clinically important adverse effects, monitoring, interactions, resistance, review of clinical studies, and guideline recommendations and clinical applications for tenofovir’s various indications.

**What Do We Already Know about This Topic?**

TDF and TAF are both recommended treatment options for HIV and hepatitis B.

**How Does Your Research Contribute to the Field?**

To our knowledge, this is the first review that summarizes and provides insight on the clinically relevant differences between TDF and TAF for all approved indications.

**What Are Your Research’s Implications toward Theory, Practice, or Policy?**

The implications of our review focus on affecting practice and highlighting relevant clinical and research information regarding TDF and TAF to provide understanding when caring for the HIV and hepatitis B population.

## Introduction

Management of HIV and AIDS has evolved substantially over the past 3 decades. As understanding of the retrovirus by the scientific community increased, advancements in its management and prevention soon followed. These advances shifted the diagnosis of HIV/AIDS from a terminal illness to a serious, but manageable, chronic medical condition.

The early part of HIV/AIDS epidemic in the United States began in 1981, after numerous cases of individuals with severe immune deficiency were reported. Six years later, zidovudine, a nucleoside reverse transcriptase inhibitor (NRTI), was the first medication approved for AIDS.^1^ Following approval of zidovudine, several other antiretroviral (ARV) agents were developed, including new classes of medications and various combinations of medications. By the mid- to late 1990s, it was realized that one active agent against HIV was insufficient to suppress viral replication and improve immune function, driving a change in management to utilization of combination therapy.^2^ Tenofovir disoproxil fumarate (TDF), an NRTI, received the Food and Drug Administration (FDA) approval for HIV in 2001, further revolutionizing disease management, serving as a critical component of backbone therapy in many HIV-positive patients.^3^

The management of hepatitis B virus (HBV) has also evolved with the progression of antiviral therapy. Significant improvements in controlling HBV and reduction of the incidence of cirrhosis and hepatocellular carcinoma due to HBV occurred over the past 2 decades. Currently, 8 medications are approved for HBV treatment.^4^ Tenofovir disoproxil fumarate received FDA approval for HBV in 2008 and is currently considered a preferred treatment option by the HBV guidelines.^3,5^

In 2015, a new formulation of tenofovir, tenofovir alafenamide (TAF), received FDA approval.^6^ Approval of TAF further transformed management of HIV and HBV, allowing for more potent nucleotide transcriptase inhibitor with a different adverse effect profile to be utilized as a mainstay of therapy.^7^

This review provides insight into the clinical pharmacology, pharmacokinetics (PK), and therapeutics of tenofovir, reviewing differences between the available formulations and considerations for selecting between TDF and TAF. A complete list of TDF- or TAF-containing regimens is provided in Table 1.

**Table 1.** FDA Approved Combination ARV Agents Containing TAF or TDF.^8–^ ^19^

Brand Name	Components	Cost Per Unit^a^
NRTI combinations		
Truvada	TDF/emtricitabine	$73.69
Descovy	TAF/emtricitabine	$73.69
Cimduo	TDF/lamivudine	$40.22
Combinations with integrase strand transfer inhibitors		
Stribild	Elvitegravir/cobicistat/TDF/emtricitabine	$135.87
Genvoya	Elvitegravir/cobicistat/TAF/emtricitabine	$129.53
Biktarvy	Bictegravir/TAF/emtricitabine	$129.53
Combinations with non-NRTIs		
Complera	Rilpivirine/TDF/emtricitabine	$117.88
Atripla	Efavirenz/TDF/emtricitabine	$119.79
Delstrigo	Doravirine/TDF/lamivudine	$84.00
Symfi/Symfi Lo	Efavirenz/TDF/lamivudine	$65.38
Combinations with protease inhibitors		
Symtuza	Darunavir/cobicistat/TAF/emtricitabine	$148.89

Abbreviations: ARV agents, antiretroviral agents; FDA, Food and Drug Administration; NRTI, nucleoside reverse transcriptase inhibitor; TAF, tenofovir alafenamide; TDF, tenofovir disoproxil fumarate.

^a^Cost is represented as the average wholesale pricing in US dollars as per RED BOOK.^20,8–18^

## Ethical Approval and Informed Consent

Ethics approval and informed consent was not required for this review of the literature.

## Pharmacology/Pharmacokinetics

Tenofovir is a nucleotide analog (NA) of adenosine 5′-monophosphate. In its parent form, tenofovir is a dianion at physiologic pH and is associated with poor membrane permeability and low oral bioavailability. To improve oral bioavailability and membrane permeability, tenofovir is commercially available as prodrugs, TDF and TAF.^21,22^

After oral administration, TDF is hydrolyzed by gut and plasma esterases to tenofovir, and TAF is metabolized mostly intracellularly by cathepsin A to tenofovir.^23,24^ Tenofovir is then activated to tenofovir–diphosphate intracellularly.^7,21^ This conversion of TDF and TAF into their active form, tenofovir–diphosphate, is similar for HIV and HBV management. Tenofovir–diphosphate works to inhibit HIV replication by competing with the natural substrate deoxyadenosine 5′-triphosphate for incorporation into DNA during HIV transcription.^21^ Tenofovir–diphosphate inhibits replication of HBV by inhibiting HBV polymerase.^25^

The prodrug TDF has demonstrated an improved PK profile and improved antiviral activity compared to the parent tenofovir in vitro and in vivo.^26,27^ In vitro, Robbins and colleagues demonstrated a greater than 100-fold increase in anti-HIV activity with TDF compared to tenofovir. They attribute this improvement to a rapid intracellular uptake of TDF, resulting in an increased intracellular accumulation of active tenofovir–diphosphate.^26^ In vivo, Gasselin and colleagues showed that single-dose oral TDF resulted in a nearly 8 times higher peripheral blood mononuclear cell exposures to tenofovir–diphosphate compared to subcutaneous tenofovir.^27^

Tenofovir disoproxil fumarate, administered as a 300-mg daily dose, has been used as a preferred backbone agent in the management of HIV for years; however, issues with bone and kidney toxicity have come to the forefront with widespread, chronic use of this medication. In phase 3 clinical trials, patients treated with TAF-containing regimens had significantly smaller mean serum creatinine increases, significantly less proteinuria, and significantly smaller decreases in bone mineral density (BMD) at the spine and hip compared to those given TDF-containing regimens.^7^ These toxicities, coupled with the need for long-term therapy with tenofovir, subsequently led to the FDA approval of TAF in 2015.

Compared to TDF, TAF has been identified as an alternative tenofovir prodrug that more efficiently loads HIV target cells, allowing for a 10-fold increased activity against HIV in vitro.^22^ In vitro, TAF is more stable in plasma and is selectively cleaved into its active metabolite intracellularly. In whole blood, TAF concentrates mainly in mononuclear cells, such as the T lymphocytes that serve as the primary site of HIV replication.^22,28,29^

After oral absorption, the majority of TDF is rapidly converted to tenofovir while in the plasma, and then intracellularly to the active tenofovir diphosphate.^6,29^ Tenofovir alafenamide, in contrast, remains stable within the plasma and is only converted intracellularly to tenofovir and then the active tenofovir diphosphate. Because of this, administration of TAF results in lower circulating plasma tenofovir levels than with TDF. These lower plasma levels of tenofovir are what lead to the differences in safety profile between TDF and TAF.^22,30^

## Adverse Effects and Monitoring

Both TDF and TAF are essential components of preferred initial HIV regimens because of their efficacy as well as improved tolerability in comparison to older agents.^31^ Common adverse effects are similar, although TDF appears to be associated with more bone and renal toxicities and TAF with increases in low-density lipoprotein (LDL) and total cholesterol.

General adverse effects for TDF may include rash, diarrhea, headache, pain, depression, asthenia, and nausea.^32^ Nucleoside reverse transcriptase inhibitors, including TDF, have been associated with severe lactic acidosis and severe hepatomegaly with steatosis.^33^ Risk factors include female sex, obesity, liver disease, and long-term therapy with an NRTI. Extra caution should be used when administering to patients with risk of liver disease, but cases have occurred in patients with no known risk factors. Patients should be monitored for any signs of an elevated lactate and hepatic function test elevations.^33^

Tenofovir disoproxil fumarate can potentially be nephrotoxic and has been associated with new or worsening renal impairment. The suspected mechanism for renal impairment with the use of TDF is damage to the proximal tubule by circulating plasma tenofovir.^34^ Tenofovir is renally eliminated via active tubular secretion as well as passive glomerular filtration. Tenofovir accumulates and causes renal damage at the proximal tubule when there is an imbalance in the process of plasma uptake and renal clearance.^22^ This manifests as a high uptake of tenofovir into the plasma, with a less rapid efflux into the urine. More severe manifestations can include renal failure or Fanconi syndrome.^33^ Risk factors for new or worsening renal impairment may include advanced HIV disease, longer treatment duration, low body weight (especially for female sex), and preexisting renal impairment.^31^

Renal function should be monitored prior to initiation and throughout therapy as clinically appropriate, and caution should be taken with administering TDF in combination with other potentially nephrotoxic agents.^34^ Per US Prescribing Information (USPI), it is recommended calculated creatinine clearance (CrCl) be determined upon initiation as well as throughout therapy as appropriate. Other measurements of renal function to monitor in situations of suspected renal impairment include serum phosphate, urine glucose, urine protein, urine phosphate, and urine calcium.^34^ Concurrent use with high-dose nonsteroidal anti-inflammatory drugs (NSAIDs) is not recommended because of specific reports of renal failure and hospitalizations in patients previously stable on TDF. Overall, concurrent use with other potentially nephrotoxic agents should be avoided if possible.

Because of its potential nephrotoxicity, TDF is to be used with caution in patients with renal impairment.^29^ Dosage adjustments to every 48 to 96 hours intervals are required if TDF is utilized with a CrCl < 50 mL/min. Tenofovir disoproxil fumarate has not been studied in patients with a CrCl < 10 mL/min.

Clinical studies of patients receiving TDF-containing regimens suggest a potential decrease in BMD from baseline.^34^ The clinical importance of these changes is unclear, but BMD assessment is recommended for both adult and pediatric patients with risk factors.^22^ Supplementation with calcium and vitamin D is likely prudent in patients receiving TDF. Patients who develop proximal tubule renal damage may be at risk of secondary osteomalacia and hypophosphatemia. These conditions should be evaluated if a patient presents with renal dysfunction and new or worsening bone symptoms. Generally, the initial decrease in BMD with ART initiation is followed by a stabilization.^31^ In comparison to TAF, BMD loss is greater with TDF.

Both adverse renal and bone effects appear to be more apparent when TDF is administered as part of a PK-boosted regimen.^31^ Frequently utilized PK boosters include cobicistat and ritonavir. The US Department of Health and Human Services (DHHS) Panel on ARV therapy recommends avoiding concomitant use of TDF with PK boosters if feasible.

As noted earlier, unique PK of TAF results in lower plasma and higher intracellular tenofovir concentrations, which allow for less bone- and kidney-related adverse effects.^26^ The most commonly reported adverse effects with TAF include abdominal and back pain, headache, fatigue, cough, and nausea.^25^ Newer data suggest that TAF may be associated with elevated lipids, fasting glucose, and increased risk of myocardial infarction, diabetes, and metabolic syndrome, compared with TDF.^35^

Unlike TDF, TAF does not require renal dose adjustment for CrCl greater than or equal to 15 mL/min, allowing for use in some renal impairment populations more so than TDF.^25,33^ Tenofovir alafenamide carries a warning for hepatic effects as well as lactic acidosis and severe hepatomegaly similar to TDF.^25^ The same risk factors and monitoring recommendations apply, and treatment should be suspected if lactic acidosis or severe hepatotoxicity develops.^25^

Despite lower risk of bone and renal toxicities, TAF has been associated with elevated LDL, high-density lipoprotein (HDL), and triglycerides in comparison to TDF.^36^ In one randomized study of TAF versus TDF, cardiovascular safety end points were monitored for 96 weeks, including fasting lipids, proportion eligible for statin therapy, cardiovascular adverse events, and estimated 10-year atherosclerotic cardiovascular disease (ASCVD) risk.^37^ No significant differences between groups were noted, except for the mean estimated 10-year ASCVD risk comparing TAF versus TDF (6.1% versus 6.2%; *P* = .04).

## Drug and Food Interactions

Unlike many other components of ARV therapy, both TDF and TAF have minimal clinically significant drug–drug interactions because of lack of CYP 450 enzymatic metabolism.^31^ Both agents are substrates of BCRP/ABCG2, and P-glycoprotein/ABCB1, and inhibitors of MRP2. Drugs that strongly affect P-glycoprotein and BCRP activity may affect TAF absorption.^25^

P-glycoprotein is an efflux pump found in intestinal tissue and functions as a biological mechanism to transport toxins out of cells.^38^ P-glycoprotein transport is infrequently a major contributor to overall drug absorption, unless the dissolution rate of the drug is very slow, or a small oral dose is given. The unique pharmacology of TAF involves a much smaller dose than is required with TDF, and it relies on metabolism intracellularly rather than primarily in the plasma, making it much more susceptible to clinically important drug interactions with P-glycoprotein manipulation. P-glycoprotein inducers will likely decrease the absorption of TAF, leading to potential treatment failure.^25^ P-glycoprotein inhibitors will lead to an increase in absorption of TAF and a higher than normal plasma concentration of the drug. Strong P-glycoprotein inducers include anticonvulsants (carbamazepine, oxcarbazepine, phenobarbital, phenytoin), antimycobacterials (rifabutin, rifampin, rifapentine), and the herbal product St. John’s wort, often used for depression. The USPI recommends against the use of these agents together with TAF because of risk of treatment failure. The exception is carbamazepine, which has undergone a drug interaction study. When utilizing carbamazepine together with TAF, the recommendation is to increase TAF to twice-daily administration instead of the standard once daily.

Because tenofovir is eliminated by the kidney, coadministration with other drugs competing for active tubular secretion may increase the plasma concentration of tenofovir and/or the coadministered drug.^25,33^ This drug interaction warning applies to both TDF and TAF. Common examples of medications that may compete for active tubular secretion in the kidney include acyclovir, cidofovir, ganciclovir, valacyclovir, valganciclovir, aminoglycosides, and NSAIDs.

The bioavailability of TDF is increased approximately 40% by a fatty meal, but this does not affect administration recommendations.^33^ Tenofovir disoproxil fumarate may be taken with or without food. Tenofovir alafenamide bioavailability is increased approximately 65% by a high-fat meal.^33^ It is recommended that TAF be administered with food.^25^

## Resistance

HIV drug resistance is caused by mutations that develop during viral replication in the setting of inadequate ARV drug exposure. When a single mutation causes resistance to other drugs in the same ARV class, this is referred to as cross-resistance. Mutations are represented by a codon number, preceded by a letter indicating the amino acid in the wild-type virus, followed by another letter indicating the amino acid substitution in the mutant virus. For example, K65R indicates that there is a lysine (K) to arginine (R) substitution at amino acid codon 65 in the reverse transcriptase enzyme.

Resistance profiles are the same for both formulations of tenofovir. However, it has been suggested that TAF may provide a higher level of protection against TDF-resistant mutant viruses due its ability to achieve higher intracellular concentrations.^39^ The primary mutation that compromises the activity of TDF and TAF is K65R. The K65R mutation is associated with cross-resistance to all other NRTIs, except zidovudine.^39-42^ The Q151M mutation alone can cause low-level resistance to tenofovir, but intermediate resistance when found in combination with other mutations.^43^ The presence of multiple thymidine analog mutations (TAMs), such as M41L, D67N, K70R, L210W, T215Y/F, and K219Q/E, can mediate tenofovir resistance.^39,44,45^ Moreover, the presence of the T69 double serine insertion mutation can further reduce the susceptibility of tenofovir in the presence of TAMs.^39,46^ Resistance to tenofovir has also been described with less common mutations such as K70E and Y115F.^47,48^

## Review of Clinical Studies

Numerous clinical studies have evaluated efficacy and safety of transitioning patients from TDF-based regimens to TAF-based regimens for both HIV and HBV.

### Tenofovir Disoproxil Fumarate Versus TAF for Management of HIV

DeJesus and colleagues designed an actively controlled, open-label, noninferiority study of virologically suppressed adult patients on 1 of 4 TDF-containing regimens. Patients were followed for at least 96 weeks and randomized to switch to a TAF-containing regimen or continue their TDF-containing regimen. The TAF regimen contained elvitegravir boosted with cobicistat and emtricitabine. Patients were randomized in a 2:1 ratio, and a total of 959 TAF patients and 477 TDF-treated patients were included for analysis.^49^

At 96 weeks, TAF demonstrated superiority over TDF, with 93% versus 89% of patients having virologic suppression of HIV RNA to < 50 copies/mL (*P* = .017). Regardless of previous treatment, the mean BMD of the hip and spine increased in the TAF group but remained stable or decreased in the TDF group (*P* < .001). Patients with spine or hip osteopenia or osteoporosis had a higher rate of recovery in the TAF group versus the TDF group. The TAF group had improved renal effects with significant improvements in proteinuria or albumin to creatinine ratios (*P* < .001). Fasting values of total cholesterol, HDL, LDL, and triglycerides were higher in the TAF group versus the TDF group, which was statistically significant, but of unknown clinical significance.^49^

The EMERALD trial investigated the efficacy and safety of switching from boosted protease inhibitors plus emtricitabine and TDF regimens to single tablet darunavir, cobicistat, emtricitabine, and TAF at 48 weeks in virologically suppressed HIV-1-infected adults. Although the study found the single-tablet regimen containing TAF to be non-inferior to boosted protease inhibitors plus emtricitabine and TDF regimens in terms of efficacy, it did find statistically significant differences in change in total fasting cholesterol, LDL-cholesterol, and total cholesterol to HDL-cholesterol ratio between the 2 study arms. Patients receiving the TAF containing regimen had significant increases in total fasting cholesterol (19.7 mg/dL versus 1.3 mg/dL, *P* < .0001), LDL-cholesterol (15.7 mg/dL versus 1.9 mg/dL, *P* < .0001), and total cholesterol to HDL-cholesterol ratio (0.2 versus 0.1, *P* = .01). Despite these increases, the clinically relevance of TAFs impact on lipids was not established. Effects on renal and bone outcomes were nonsignificant; however, patients treated with TAF had preservation of estimated glomerular filtration rate (eGFR), less tubular proteinuria, and improvements in BMD scores compared to TDF-treated patients.^35^

Sax and colleagues evaluated the safety and efficacy of TAF as part of elvitegravir/cobicistat/emtricitabine versus TDF as part of elvitegravir/cobicistat/emtricitabine in a randomized, multicenter trial. Results at week 48 demonstrated high rates of viral suppression among both groups (88.4% versus 87.9%) with similar improvements in CD4 count. Similar to previous studies, patients receiving TAF had smaller reductions in estimated CrCl (−5.5 mL/min versus −10.1 mL/min, *P* = .041), significantly less proteinuria, and smaller changes in BMD for hip (−0.62% versus −2.39%, *P* < .001) and spine (−1.00% versus −3.37%, *P* < .001).^50^ At 96 weeks, 86.6% in the TAF arm and 85.2% in the TDF arm were virally suppressed (difference 1.5%; 95% confidence interval [CI]: −1.8% to 4.8%)]. Smaller declines in BMD and more favorable changes in proteinuria and albuminuria continued to be observed in TAF-treated patients.^51^ At 144 weeks, TAF was superior to TDF in virologic efficacy (84.2% versus 80.0%; 95% CI: 0.6%-7.8%). Tenofovir alafenamide also continued to have less impact on BMD and renal biomarkers compared to TDF.^36^

The ADVANCE trial is an ongoing, phase III, open-label, randomized trial conducted in South Africa in which dolutegravir plus emtricitabine plus TDF or TAF were compared to standard of care for the treatment of HIV. Over 1000 participants were enrolled and followed for 96 weeks. Results at week 48 showed viral suppression to an HIV-1 RNA level of <50 copies/mL in 84% in the TAF group and 85% in the TDF group compared to 79% in the standard-of-care group, demonstrating non-inferiority of both tenofovir-based regimens. The TAF-based regimen had less effect on bone density and renal function than the other regimens. Notably, weight increase was greatest in the TAF-based group and among female patients.^52^

In a recent abstract by Surial and colleagues, 3430 patients from the Swiss HIV cohort study receiving TDF- or TAF-containing ARV therapy were followed for changes in renal function. If baseline eGFR was ≥90 mL/min, after 18 months eGFR trajectories were similar between the TDF and TAF groups (predicted difference in eGFR: 0.3 mL/min, 95% CI: 1.5-2.0 mL/min). If baseline eGFR was <60 mL/min, difference in eGFR at 18 months was 9.6 mL/min (95% CI: 5.1-14.0 mL/min) between patients receiving TAF compared to those receiving TDF. The authors concluded that there was an increase in eGFR over time in TAF compared to TDF in patients with moderate-to-severe renal impairment.^53^

In a recently published meta-analysis of 11 randomized clinical trials, Hill and colleagues sought to investigate if the higher risk of renal and bone adverse effects seen with TDF in comparison to TAF was associated with the concurrent use of the PK boosters ritonavir or cobicistat, rather than TDF’s higher plasma concentration. They also sought to investigate any differences in efficacy of viral suppression between TAF and TDF with and without PK boosting. Nine of the reviewed clinical trials included in the aforementioned meta-analysis were studied in an HIV-1 population and 2 in HBV. The 11 trials consisted of over 4500 patients receiving boosted regimens and over 3500 patients receiving unboosted regimens. Participants across these trials were predominantly male (83%), white (59%), and had a mean age of 41 years.^54^ Of note, no direct studies of unboosted TAF versus unboosted TDF have been conducted to date.

Results from the meta-analysis demonstrated that patients taking boosted TAF had 2% higher rates of HIV RNA suppression <50 copies/mL in comparison to boosted TDF (95% CI: 0%-4%, *P* = .05). No significant differences in HIV RNA suppression were observed in those taking unboosted regimens. Discontinuation secondary to renal adverse events was 1% lower in patients receiving boosted TAF versus boosted TDF (95% CI: −1% to 0%, *P* = .002).^54^

The risk of bone fractures with boosted TAF compared to boosted TDF was 1% lower (*P* = .04). Patients taking boosted TAF were significantly less likely to stop treatment secondary to bone adverse effects than those taking boosted TDF (*P* = .03). No differences in risk of fractures or bone-related adverse events between unboosted TDF and TAF were detected.^54^

Patients with boosted TDF showed a statistically significant lower rate of HIV RNA suppression of <50 copies/mL (*P* = .05), as well as larger decreases in BMD (*P* = <.001), more bone fractures (*P* = .04), and more discontinuations for bone (*P* = .03) or renal (*P* = .002) adverse events. There were no significant differences in HIV RNA suppression rates when comparing unboosted TDF and unboosted TAF.^54^

This meta-analysis highlights that the differences in TDF and TAF safety profile may have less to do with the formulations themselves, and more so when combined with PK boosters that further increase the drug’s area under the curve. When looking at initial recommended regimens for ART, PK boosters are generally not recommended together with TDF or TAF. These data provide some indication of the safety of utilizing TDF, especially if no PK booster is combined.^54^

### Tenofovir Disoproxil Fumarate Versus TAF for Management of HIV Pre-Exposure Prophylaxis

Tenofovir disoproxil fumarate in combination with emtricitabine was the initial formulation of tenofovir FDA approved for HIV pre-exposure prophylaxis (PrEP). Recently released data from the DISCOVER trial support the use of TAF in combination with emtricitabine as an effective and safe means of PrEP, leading to its FDA approval for HIV PrEP in certain patient populations. The DISCOVER trial is a phase III, randomized, parallel, double-blind study evaluating the safety and efficacy of fixed-dose emtricitabine (F) and TAF for PrEP in men and transgender women who have sex with men and are at risk of HIV-1 infection. The primary outcome of the study is the incidence of HIV-1 infection per 100 person-years in patients who receive F/TAF versus F/TDF. Secondary outcomes include changes in BMD, renal function, and development of ARV resistance.^55^ The study enrolled over 5000 participants at risk of HIV acquisition, half received daily F/TAF and the other half received daily F/TDF. Participants were followed up for up to 96 weeks. In total, 22 new HIV infections occurred over the course of the study, 7 in the F/TAF group, and 15 in the F/TDF group, demonstrating the non-inferiority of F/TAF compared to F/TDF for HIV PrEP. Both regimens were well tolerated; however, F/TAF had significantly better bone and renal safety outcomes compared to F/TDF.^56^

### Tenofovir Disoproxil Fumarate Versus TAF for Management of HBV

Recently, 96-week data from 2 ongoing international, phase III, randomized, double-blind trials evaluating the safety and efficacy of TAF versus TDF for the treatment of chronic HBV infection were released. The trials enrolled both treatment-naive and treatment-experienced patients, with and without positive hepatitis B envelope antigen (HBeAg), randomizing patients in a 2:1 ratio to receive TAF 25-mg orally once daily or TDF 300 mg orally once daily. In total, 866 patients received TAF and 432 received TDF. At week 96, rates of viral suppression were similar in HBeAg-positive patient receiving TAF and TDF (73% versus 75%, 95% CI: −8.3% to 3.9%; *P* = .47) and in HBeAg-negative patients receiving TAF and TDF (90% versus 91%, 95% CI: −7.0% to 5.8%; *P* = .84). The study concluded that TAF remained as effective as TDF in suppressing HBV replication over the 2-year treatment period, without development of virologic resistance.^57^

Additionally, TAF was associated with significantly higher resolution of elevated ALT at week 96 of treatment, significantly smaller decreases in BMD in the hip (mean % change −0.33% versus −2.51%) and lumbar spine (mean % change −0.75% versus −2.57%), and a significantly smaller changes in eGFR (−1.2 mg/dL versus −4.8 mg/dL).^57^ These findings demonstrate the continued safety of TAF compared to TDF.

## Guidelines, Recommendations, and Clinical Application for the Use of TDF and TAF (HIV, PrEP, PEP, and HBV)

### Guidelines, Recommendations, and Clinical Application for the Use of TDF and TAF in HIV

Nucleoside reverse transcriptase inhibitors are essential constituents that make up the backbone of a complete ARV regimen. As per the Guidelines for the Use of Antiretroviral Agents in Adults and Adolescents Living with HIV, an ARV regimen for treatment-naive patients should consist of 2 NRTIs in combination with a third active drug from one of the following classes: integrase strand transfer inhibitor, non-NRTI, or protease inhibitor with a PK enhancer. Of the 7 US FDA-approved NRTIs for HIV treatment, tenofovir is a primary agent recommended by the DHHS.^31^

Tenofovir alafenamide or TDF in combination with emtricitabine or abacavir plus lamivudine are the preferred NRTI combinations in initial ARV regimens.^31^ Safety, cost, and access are some factors to consider when choosing between the 2 formulations of tenofovir. As discussed previously, TAF has fewer bone and renal toxicities when compared to TDF, while TDF is associated with lower lipid levels.^31^ Both formulations of tenofovir are available in different combination tablets for ease of administration. Table 1 includes a comprehensive list of available FDA-approved combination ARV agents containing TAF or TDF and their costs in the United States.

Of note, the World Health Organization currently does not recommend TAF-based regimens for use in HIV treatment due to gaps in data. These gaps include safety of use in pregnant women and lack of experience in patients receiving concurrent treatment for tuberculosis.^58^ Additionally, as seen in the ADVANCE trial, there is an increased risk of developing clinical obesity when TAF is used in combination with dolutegravir.^52^

### Guidelines, Recommendations, and Clinical Application for the Use of TDF and TAF in PrEP

Pre-exposure prophylaxis encompasses the use of ARV agents in reducing the risk of acquiring HIV infection in high-risk individuals. The Centers for Disease Control and Prevention (CDC) endorses the use of once-daily oral TDF or TAF and emtricitabine, in conjunction with patient counseling and monitoring, to prevent new HIV infections in adults who are at substantial risk.^59^ The 2017 CDC guidelines recommend PrEP for adults who meet the following criteria: sexually active adult men who have sex with men (MSM), adult heterosexually active men and women who do not regularly use condoms with partners of unknown HIV status who may be at high risk of contracting HIV, and adult persons who inject drugs (PWID).^59^

Truvada, a co-formulated tablet of TDF 300 mg and emtricitabine 200 mg, is FDA approved for the indication for PrEP in adults and adolescents weighing at least 35 kg.^60^ Tenofovir disoproxil fumarate alone may be considered an alternative regimen in heterosexually active adults and PWID, but not for MSM, since efficacy has not been studied in this population.^59,61,62^

Results from a phase 1 PK study showed TAF exhibited lower mucosal tenofovir concentrations compared with TDF, which led to concerns that TAF may be less effective for PrEP.^62,63^ However, more recent data from the phase 3 DISCOVER study showed non-inferiority of TAF to TDF when used in combination with emtricitabine for PrEP in cisgender MSM and transgender women.^56^ Data from this trial led to the FDA approval of TAF in combination with emtricitabine for PrEP.^35^

### Guidelines, Recommendations, and Clinical Application for the Use of TDF and TAF in PEP

Postexposure prophylaxis (PEP) with ARV agents is warranted in individuals after a potential exposure to HIV in order to prevent becoming HIV-infected. A 28-day course of PEP should be initiated within 72 hours to HIV-negative persons after an exposure to blood, genital secretions, or other potentially infected body fluids of persons known to be HIV-infected or of unknown HIV status.^64^ Unlike PrEP, there are insufficient data to recommend a specific combination of ARV agents as the most effective regimen in PEP.

Based on evidence demonstrating maximal viral suppression in the treatment of HIV when at least 3 ARV drugs are used, the CDC recommends a 3-drug PEP regimen in order to prevent becoming HIV-infected after a potential exposure. In adults and adolescents at least 13 years old with normal renal function (CrCl >60 mL/min), a 3-drug regimen consisting of TDF 300 mg co-formulated with emtricitabine 200 mg (Truvada) once daily with raltegravir 400 mg twice daily or dolutegravir 50 mg once daily is preferred.^64^ A 3-drug regimen consisting of TDF, emtricitabine, and raltegravir dosed based on body weight is preferred for PEP in children 2 to 12 years old.

### Guidelines, Recommendations, and Clinical Application for the Use of TDF and TAF in HBV

Both TDF and TAF are FDA-approved and preferred therapies in treatment of chronic HBV in both treatment-naive and treatment-experienced patients.^5^ Compared to other available HBV therapies, TDF and TAF demonstrate increased HBV-DNA suppression, regardless of the HBeAg status of the patient and maintain a higher barrier to resistance.^5^ In the management of antiviral resistance, TDF has been shown to remain effective in HBV resistant to other NAs such as lamivudine, adefovir, and entecavir.^65-67^ Tenofovir disoproxil fumarate is the preferred salvage therapy, particularly in patients in whom the history of past NAs is unclear.^5^ In patients with HBV coinfected with HIV, tenofovir (TDF or TAF) plus lamivudine or emtricitabine is recommended.^31^ Although there is more experience with TDF compared with TAF in HBV, TAF appears to be equally effective and is associated with less renal and bone toxicity.^20,68^

## Conclusion

Both TDF and TAF serve as vital components in preferred treatment regimens for HIV and HBV. The agents have similar efficacy and unique adverse effect profiles. The main pharmacologic differences between the 2 formulations of tenofovir are decreases in renal and bone adverse effects, and increases in total cholesterol and LDL observed with TAF. The increased rate of bone and kidney adverse effects associated with TDF was attributed to its use in combination with a PK booster. When utilized without a PK booster, TDF’s renal and bone effects may be similar to TAF, although no clinical studies comparing TAF and TDF in this setting have been conducted. Due to the nature of HIV and HBV management requiring chronic lifelong treatment, the choice between TAF or TDF should be based on patient specific factors, concomitant use of other ARV agents, and cost.
